# Inter- and Intraobserver Repeatability of Myocardial Flow Reserve Values Determined with SPECT Study Using a Discovery NM530c Camera and Corridor 4DM Software

**DOI:** 10.3390/jpm11111164

**Published:** 2021-11-09

**Authors:** Paweł Cichocki, Michał Błaszczyk, Kamila Cygulska, Krzysztof Filipczak, Zbigniew Adamczewski, Jacek Kuśmierek, Piotr Lipiec, Jarosław Damian Kasprzak, Anna Płachcińska

**Affiliations:** 1 Department of Nuclear Medicine, Medical University of Lodz, 92-216 Lodz, Poland; pawel.cichocki@umed.lodz.pl (P.C.); jacek.kusmierek@umed.lodz.pl (J.K.); 2 Department of Quality Control and Radiological Protection, Medical University of Lodz, 92-216 Lodz, Poland; michal.blaszczyk@umed.lodz.pl (M.B.); krzysztof.filipczak@umed.lodz.pl (K.F.); anna.plachcinska@umed.lodz.pl (A.P.); 3 Chair and Department of Cardiology, Bieganski Hospital, Medical University of Lodz, 91-347 Lodz, Poland; cygulskakamila@gmail.com (K.C.); lipiec@ptkardio.pl (P.L.); kasprzak@ptkardio.pl (J.D.K.)

**Keywords:** coronary artery disease, discovery NM530c, myocardial flow reserve, myocardial perfusion imaging, technetium Tc 99m sestamibi

## Abstract

Background: Myocardial blood flow (MBF) and flow reserve (MFR) examination, especially useful in the diagnosis of multivessel coronary artery disease (CAD), can be assessed with a cadmium-zinc-telluride (CZT) SPECT gamma camera, as an alternative to the expensive and less available PET. However, study processing is not free from subjective factors. Therefore, this paper aims to evaluate intra- and interobserver repeatability of MBF and MFR values obtained by the same operator and two independent operators. Methods: This study included 57 adult patients. MBF and MFR were assessed using a Discovery NM530c camera in a two-day, rest/dipyridamople protocol, using ^99m^Tc-MIBI. Data were processed using Corridor4DM software, twice by one operator and once by another operator. Results: The repeatability of the assessed values was quite good in the whole myocardium, LAD and LCX vascular territories, but was poor in the RCA territory. Conclusions: The poor repeatability of MBF and MFR in RCA vascular territory can be explained by poor automatic orientation of the heart axis during post-processing and a so-called “cardiac creep” phenomenon. Better automatic heart orientation and introduction of automatic motion correction is likely to drastically improve this repeatability. In the present state of the software, PET is better for patients requiring assessment of MFR in the RCA territory.

## 1. Introduction

Assessment of the absolute myocardial blood flow (MBF) and calculation of the myocardial flow reserve (MFR) are among the newest diagnostic methods used in nuclear cardiology. In contrast to the determination of the fractional flow reserve (FFR) in coronary angiography, which allows the assessment of the hemodynamic significance of epicardial coronary artery stenosis, the MFR allows the assessment of the coronary reserve of the entire left ventricular myocardium and selected vascular territories. An additional advantage of this method is its non-invasive character. This broadens the diagnostic potential of myocardial perfusion imaging (MPI), particularly in the diagnosis of multivessel coronary artery disease (CAD), where standard perfusion study is less sensitive. MFR assessment with cadmium-zinc-telluride (CZT) semiconductor gamma cameras is an alternative to PET examinations using ^82^Rb, ^15^O or ^13^N radionuclides, which are expensive, have very short half-lives and, apart from ^82^Rb, are practically not used in routine clinical diagnosis. Owing to a recent ASNC/SNMMI position statement [1], severely reduced global hyperemic MBF and MFR can be used to identify patients at high risk for major adverse cardiovascular events, including death. Although thresholds may vary in different labs using different software, in general, an MFR of less than 1.5 should be considered a high-risk feature on MPI PET and is associated with increased likelihood of a multivessel obstructive CAD. A severe reduction in hyperemic MBF (<1.5 mL/min/g) or MFR (<1.5) in a single vascular territory in a patient with normal PET myocardial perfusion imaging results by semiquantitative visual analysis should raise the possibility of flow-limiting CAD.

However, processing of the data obtained in this study (both in SPECT and PET) is not free from subjective factors and must be performed with the utmost care. For the correct calculation of MFR, it is necessary to align the heart images in the rest and stress studies in the appropriate axes. Moreover, as some authors emphasize, the quality of the study is influenced by artifacts resulting from the movement of the heart during the study (the so-called “cardiac creep” phenomenon—a change in the heart position, probably caused by faster and deeper breathing in response to dipyridamole) [2,3]. Therefore, it is also necessary to perform motion correction while processing the study. Due to the presence of subjective factors in data processing, it is necessary to assess the reliability of the MBF and MFR values by assessing their inter- and intraobserver repeatability. Such an analysis was carried out for the PET MFR studies [4], but so far, there are no comprehensive reports on the repeatability of the values of these parameters obtained using SPECT cameras, except for brief mentions in a few articles [5,6].

The aim of this work is to assess the repeatability of MBF and MFR determination in studies carried out with technetium-99m-labeled sestamibi (^99m^Tc-MIBI) using the SPECT CZT Discovery NM530c gamma camera, as a continuation of our earlier, preliminary report [7].

## 2. Materials and Methods

A total of 64 adult patients with symptoms of CAD and no history of coronary artery bypass graft (CABG), who were planned for invasive coronary angiography, were enrolled in the study. Each patient underwent MPI with MFR assessment. Seven patients were excluded after quality control of the acquired dynamic images (due to artifacts caused by significant patient motion or irregular bolus time-activity curves). The remaining 57 patients were included in the study. Their basic demographic and clinical data are presented in Table 1 and Table 2.

All patients underwent invasive coronary angiography, which revealed critical stenosis of at least one coronary artery in 23 patients (including 8 patients with two-vessel disease and 2 patients with three-vessel disease). Critical stenosis affected the left anterior descending (LAD) artery in 14 cases, the left circumflex (LCX) artery in 11 cases and the right coronary artery (RCA) in 10 cases. There were no findings of critical stenosis in the coronary arteries of the remaining 34 patients. The time span between MPI and coronary angiography did not exceed 3 months.

### 2.1. Patient Preparation and Data Acquisition

Prior to the study, each patient followed the routine protocol of preparation for the MPI performed with the dipyridamole stress test. Treatment with drugs that may interfere with the MPI was suspended for an appropriate period prior to the study. Calcium antagonists and nitrates were discontinued 2 days prior to the studies, and trimetazidine and its derivatives were discontinued for 7 days. In addition, patients were recommended to discontinue medication and avoid beverages and foods containing methyloxnatines (such as caffeine or theophylline) for 2 days prior to the dipyridamole pharmacological test. Patients were also asked to eat a sandwich with cheese or a hard-boiled egg immediately prior to the study, to accelerate hepatic clearance of the radiopharmaceutical agent.

MFR was assessed using a CZT Discovery NM530c camera (GE Healthcare, Chicago, IL, USA) in a two-day protocol—at rest and after the pharmacological test with dipyridamole. Each time the patient was first administered intravenously 37 MBq of ^99m^Tc-MIBI (for positioning on the camera, with the heart in the center of the field of view of detectors) followed by 550 MBq in a quick bolus injection, carried out at the same time as the start of image acquisition. Dynamic image acquisition continued for 8 min. The stress part of the study consisted of a pharmacological test with dipyridamole, administered intravenously (dose: 0.56 mg/kg), after positioning the patient on the camera. The radiopharmaceutical bolus was administered 3 min after the injection of dipyridamole.

### 2.2. Data Processing

**Figure 1:**
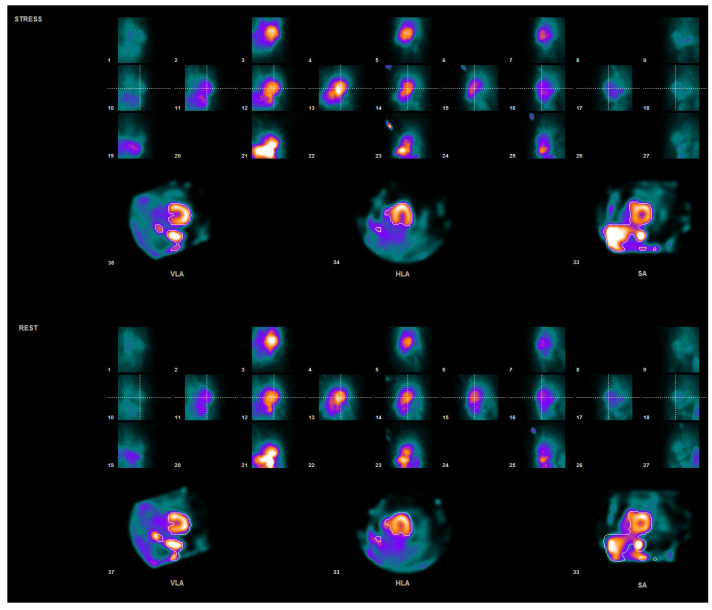
Quality control of patient positioning.

Data acquired using the gamma camera included dynamic images in listmode format, without attenuation correction. The dynamic data were reframed into 23 frames (15 × 6 s, 4 × 30 s, 4 × 60 s) and reconstructed according to the standard protocols of the camera supplier. Before further evaluation, a quality control of the patient positioning on the camera was carried out, by making sure that the heart is in the center of the field of view of the detectors and in the same position in both parts of the study (stress and rest)—Figure 1.

**Figure 2:**
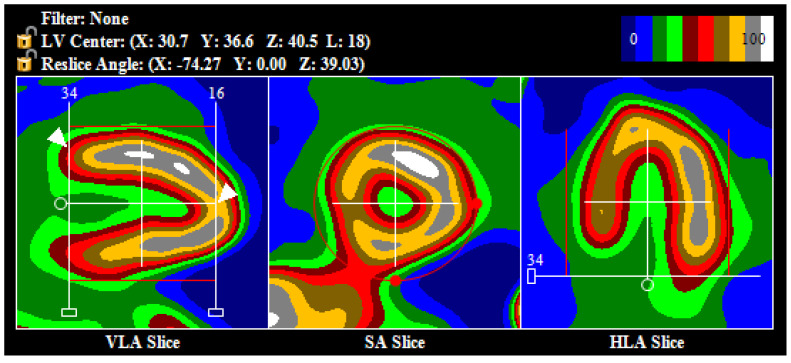
Proper orientation of image axis (along the long axis of the left ventricle) and first and last slice (white arrows), with a correct setup of the mask (red lines), cutting off extra-cardiac activity. Abbreviations: VLA—vertical long axis, SA—short axis, HLA—horizontal long axis.

SPECT images reconstructed in this way were processed using Corridor 4DM software, ver. 2015.0.2.66 (INVIA, Ann Arbor, MI, USA), validated on the Discovery NM530c camera using 4DM with ^86^Rb or ^13^N ammonia PET MBF as a reference standard [8], according to which non-attenuation corrected one-tissue compartment model can be used for clinical purposes on this camera. Processing consisted of aligning the image axes with the long axis of the left ventricle (LV), such that the anterior slice crosses the center of the apex of the heart and the posterior slice is located at the base of LV, at a point where the anterior wall activity is about 50% of the maximum (at the border between light red and dark red on a 10-step color scale) and adjusting the mask to cut off extra-cardiac activity (Figure 2).

**Figure 3:**
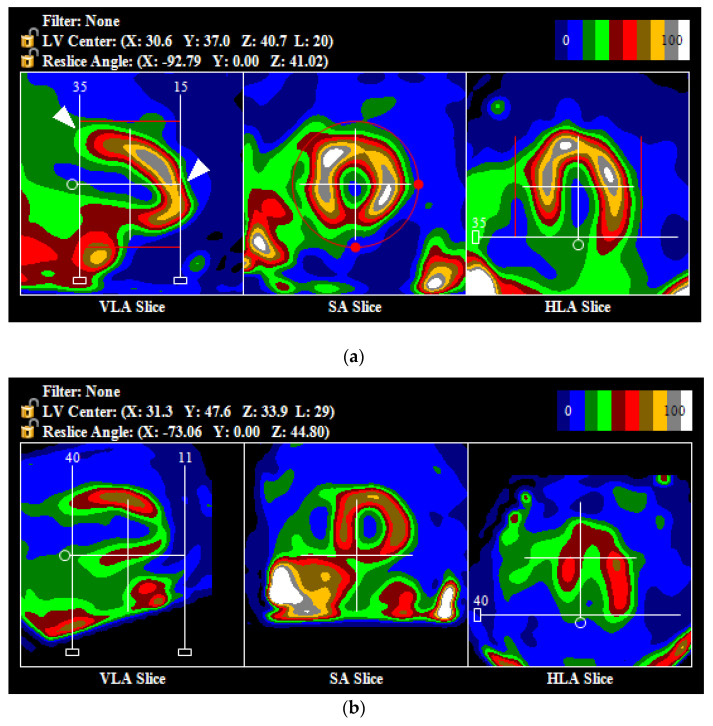
Examples of incorrect automatic setup of the axis of the images (not aligned with the long axis of the left ventricle) and wrong position of the first and last slice (white arrows) (**a**) and incorrect automatic set-up of the center of the axis, which was positioned below the left ventricle (**b**). Abbreviations: VLA—vertical long axis, SA—short axis, HLA—horizontal long axis.

Initial post-processing was done automatically. Then, if necessary, it was adjusted manually. At this stage, each rest and stress study was assigned a numerical value, depending on the quality of the automatic image orientation: 0—automatic processing, which required little to no modification (Figure 2), 1—the central point of the axis was positioned correctly, at the center of the LV, but the angle of the image axis needed to be adjusted (Figure 3a), 2—the center of the axis was positioned incorrectly, outside of the LV center (Figure 3b).

In the next step, MBF and MFR values were generated using the one-tissue-compartment model with empirical Renkin-Crone compensation for the low first-pass extraction of the radiopharmaceutical and absence of attenuation correction [8], applying manual motion correction. Attenuation correction was not applied.

To assess repeatability, processing of each study was performed twice by one operator (with a two-week interval between each processing) and once by another, less experienced operator.

### 2.3. Statistical Analysis

Normality of the distributions was tested with a Shapiro–Wilk test. The repeatability of the examined parameters, some of which were not distributed normally, was assessed using the non-parametric Spearman’s rank correlation coefficient and the r^2^ determination coefficient, as well as—for selected parameters—Bland–Altman plots. The F-test was used to assess the relationship between standard deviations used to draw Bland–Altman plots. In all analyses, statistical significance was considered to be achieved when *p* ≤ 0.05. The calculations were carried out using Statistica v13.1 (StatSoft Polska, Kraków, Poland) and LibreOffice v7.2 (The Document Foundation, Berlin, Germany) software.

## 3. Results

### 3.1. Myocardial Blood Flow

The repeatability of the MBF values obtained by the same operator with a two-week interval between assessments and by two different operators was assessed in the whole myocardium of the LV (TOT) and in the vascular territories of the major coronary arteries (LAD, LCX and RCA). Spearman’s rank correlation coefficients were used as a measure of repeatability (Table 3).

MBF values in the RCA vascular territory consistently showed statistically significantly weaker correlations than in other territories, both in assessments carried out by one and two operators. Additionally, in the RCA territory, the correlations of MBF results obtained by the same operator were significantly stronger than for two independent operators (0.94 vs. 0.88 *p* = 0.075). In the whole myocardium as well as LAD and LCX territories, the differences between the correlations of the results obtained by the same and two different operators were statistically insignificant.

Since the correct initial, automatic orientation of the images practically eliminates the subjective factor at the first stage of the study processing, the impact of its quality on the repeatability of the obtained results was assessed. The number of rest and stress studies with different grades of automatic image alignment quality is summarized in Table 4. Manual adjustments were required in 75% of all 114 studies (including 91% of the rest studies and 60% of the stress studies).

The repeatability of the MBF values obtained by the same and two independent operators in the rest and stress studies that did not require significant manual adjustments (quality of automatic orientation marked as “0”) was compared with studies that required corrections (marked as “1” or “2”) (Table 5). In the group that required manual corrections of the image orientation and axis center, the repeatability of the MBF values was significantly lower in case of RCA vascular territory, both in assessments by the same (*p* = 0.0024) and two different operators (*p* = 0.0086), and in case of the LAD territory and the whole myocardium, only in assessments by the same operator (*p* = 0.04 and *p* = 0.0005, respectively). In the remaining assessments, the differences were not statistically significant.

### 3.2. Myocardial Flow Reserve

**Figure 4:**
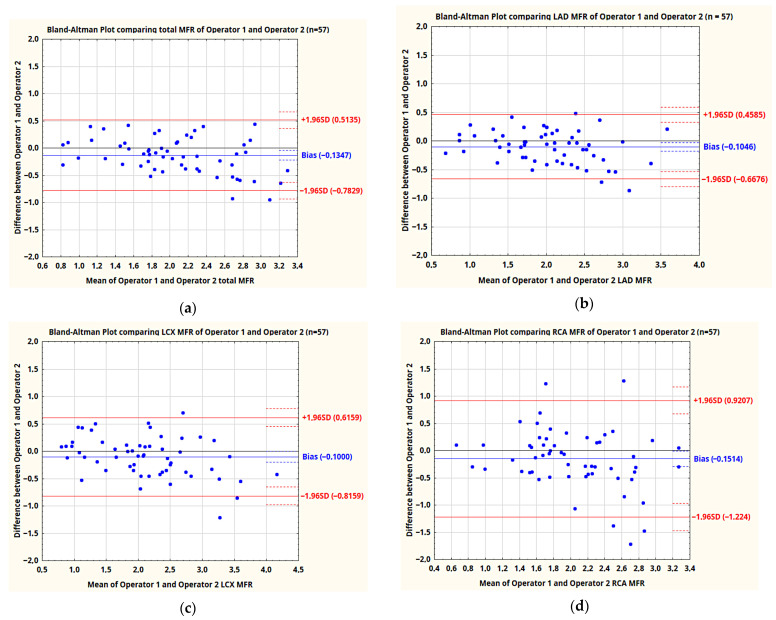
Bland–Altman plots comparing MFR values obtained by two operators in the whole LV—TOT (**a**) and three vascular territories—LAD (**b**), LCX (**c**) and RCA (**d**).

Repeatability of MFR values was assessed similarly to MBF. Correlation coefficients of the results obtained by the same and two different operators are presented in Table 6, supplemented by the mean differences and standard deviations between the results obtained by two operators. In addition, Bland–Altman plots comparing MFR values obtained by two independent operators were plotted (Figure 4a–d).

In the assessments by two independent operators, as in the case of MBF, the values of MFR in the RCA vascular territory showed a statistically significantly weaker correlation compared to the MFR values in the whole LV as well as LAD and LCX vascular territories. In the RCA territory, correlation of MFR values obtained by the same operator was also significantly stronger than for two independent operators (0.84 vs. 0.67–*p* = 0.035), with insignificant differences in the whole myocardium and LAD and LCX territories.

The number of patients in whom automatic image alignment was correct in both rest and stress studies (five patients) is not sufficient to perform a statistical analysis of repeatability of MFR values in this group.

## 4. Discussion

A Discovery NM530c CZT gamma camera allowed for the assessment of MFR, which is a valuable addition to non-invasive MPI studies and can address some of their weaknesses, namely their lower sensitivity in the diagnosis of multi-vessel CAD, especially in cases of three-vessel disease. However, this dynamic examination, both in SPECT and PET techniques, is prone to errors and must be performed with the utmost care. There are several potential sources of such errors, both at the image acquisition stage and during post-processing.

Positioning of the patient on the camera, respiratory motion and patient movement can significantly affect the results of the study, both in terms of MFR values and the perfusion evaluation [9,10,11,12]. Fast imaging times minimize the incidence and impact of artifacts due to patient movement. Respiratory motion remains an important factor, but it can be corrected during study processing if necessary. On the other hand, proper patient positioning on the Discovery NM530c camera is essential due to the setup of CZT detectors and the use of pinhole collimators, which limit the effective field of view of the camera. Patients who cannot be positioned correctly, e.g., because of severe obesity, are not eligible for MPI studies using this camera.

During data post-processing, there are also two stages that are prone to error due to subjective factors. The first is manual reorientation of the images to match the long axis of LV. Corridor 4DM software automatically adjusts the position and angle of the image axis, which should minimize the need for manual corrections. However, in this study, automatic orientation required significant manual adjustments, both in terms of image alignment and positioning the center of the axis at the center of the LV cavity, in the majority of cases (75% of all studies, including 91% of rest studies and 60% of stress studies). In PET studies, such problems are not reported frequently—automatic orientation of the images in most cases does not require such significant corrections, which can be indirectly deduced from the excellent repeatability of MBF values reported in PET studies (r^2^ = 0.99, [4]), despite performing manual motion correction, as in our study. It is not clear why such differences exist. The likely reasons can be seen in the different characteristics of the images acquired on the Discovery NM530c camera. The use of pinhole collimators causes heterogeneous image acquisition (enhanced in the center of the field of view and weakened on the periphery) and requires different reconstruction algorithms. This may result in a different distribution of extra-cardiac activity on images, compared to PET (especially the sub-diaphragmatic activity appearing at the edge of the field of view at the end of the dynamic study, most likely resulting from the presence of the radiopharmaceutical in the blood pool in abdominal tissues and organs), which may hinder automatic detection of the heart. We observed that when the center of the image axis was mispositioned (placed outside of the LV), it was most often shifted down, towards the activity located under the diaphragm. Moreover, the image of the heart on transversal slices is located closer to the image center than in the PET studies, which may also cause a different outcome of the automatic orientation of the images.

As demonstrated by Monroy-Gonzalez et al., MBF and MFR values may significantly differ depending on the software used to process the study [13]. It is possible that this is due to a different reliability of the protocols responsible for automatic image orientation and motion correction, which could require more or less manual corrections depending on the program. The Corridor 4DM software is constantly being updated and it is to be expected that the problem with incorrect automatic image orientation will soon be solved, which should definitely improve the repeatability of the MBF and MFR results. As demonstrated in this study, incorrect automatic image alignment significantly decreases the repeatability of MBF values, especially in the area of the RCA vascular territory. Since MBF values from the rest and stress studies are the basis for the calculation of MFR, it can be expected that lower repeatability of MBF results in either of these studies will have a negative impact on the repeatability of MFR values as well. In this study, the group of patients with good automatic image orientation in both stress and rest studies was too small to perform a reliable statistical analysis. However, taking into account that incorrect automatic image orientation mainly affects the MBF in the RCA vascular territory, it can, at least in part, explain the significantly weaker repeatability of MFR values in this area achieved in our study.

On the other hand, it should be emphasized that in the stress and rest studies, in which the automatic image orientation required little to no manual adjustments, the correlation of all MBF values was very high. The less reliable automatic image orientation is one of the most significant differences in the processing of studies performed using the CZT camera compared to PET, so it is likely that this aspect is partly responsible for the weaker repeatability of our results compared to analogous reports for PET studies [4].

Manual motion correction is the final stage in study processing, which is influenced by subjective factors. This is an essential part of the MFR assessment and has a significant impact on the results, especially affecting the RCA vascular territory. This effect is attributed to the “cardiac creep” phenomenon, which is a movement of the heart that is presumed to be caused by faster and deeper breathing following administration of dipyridamole. This motion may cause the blood pool inside the LV to overlap with the inferior wall contour, which has a significant impact on MBF and MFR values in the corresponding RCA territory. Due to this, MFR values in the RCA territory are the most susceptible to variability resulting from subjective, manual motion correction [2,3].

**Figure 5:**
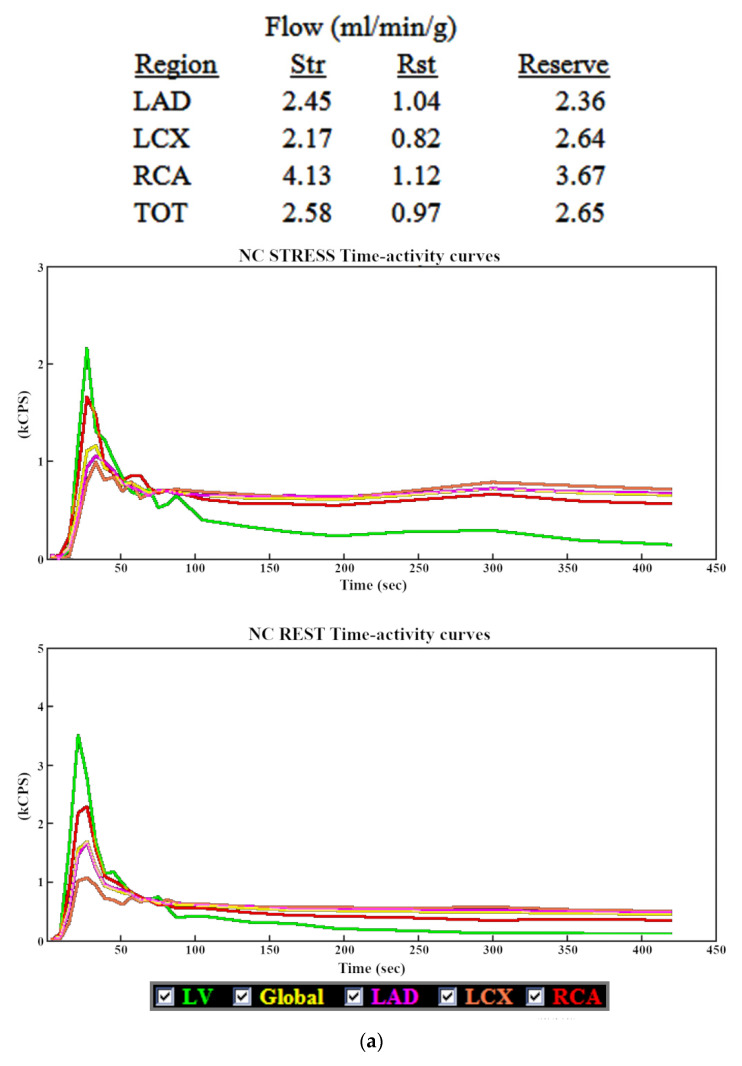

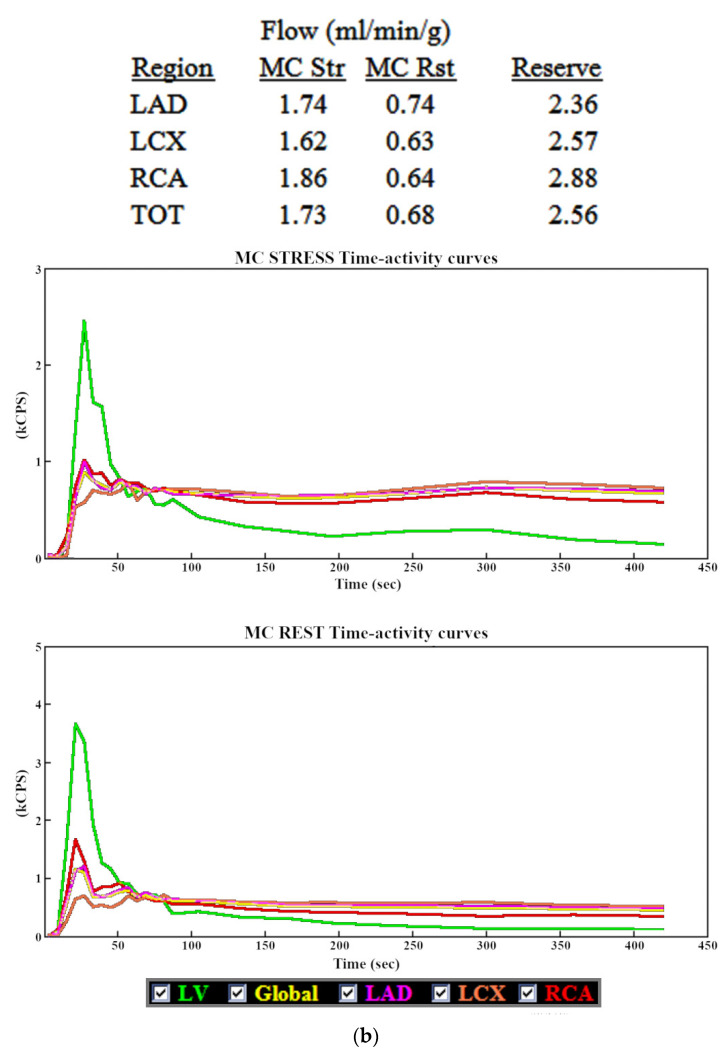
Example of MBF and MFR results with time-activity curves before (**a**) and after (**b**) motion correction, with a significant difference in the RCA vascular territory.

The effect of errors related to heart motion can be illustrated by the example shown on Figure 5. The time-activity curve in the RCA territory (red) before motion correction is significantly higher than curves in other vascular territories. This is especially evident in the stress study, where the RCA curve is almost as high as the “bolus” curve (green), generated from the ROI placed in the projection of the mitral valve. This is most likely the result of inferior wall contour (generated using the 60 s frames from the last part of the study) overlapping with the LV cavity, due to the upward movement of the heart during image acquisition. In the presented case, manual motion correction had a significant impact on the MFR in RCA territory, while the values in other territories as well as the MFR of the whole LV were affected to a much lesser extent. Therefore, it can be expected that the greater impact of subjective, manual motion correction on the values in RCA territory will negatively affect their repeatability in this area, which was demonstrated in our study.

It should also be mentioned that the lower extraction coefficient of ^99m^Tc-labeled radiopharmaceuticals (^99m^Tc-MIBI in this case) compared to ^82^Rb or ^13^N [14] can be an additional source of error, since it requires multiplying the raw blood flow results by appropriate normalization values (Renkin-Crone compensation) and results in the multiplication of random measurement errors as well.

The above factors suggest that in the current version of the Corridor 4DM software, the MFR evaluation with the CZT Discovery NM530c camera with manual motion correction has a fairly high repeatability in case of the whole LV myocardium, as well as regional MFR of LAD and LCX vascular territories. However, in the RCA territory, the repeatability is significantly lower, especially in the case of assessments carried out by two independent operators.

Automatic motion correction software has recently been developed and has shown promising results in PET studies [4]. It is reasonable to assume that its implementation in SPECT studies should also improve the reliability of the MFR assessments, which is necessary for the evaluation of MFR in the RCA territory. However, this software is not implemented in our current version of the Xeleris workstation (4.0). Therefore, the comparison of intra- and interobserver repeatability of MFR values obtained with manual and automatic motion correction will be the subject of future research.

## 5. Conclusions

Our analysis of dynamic SPECT studies of myocardial blood flow and flow reserve, performed using the Discovery NM530c gamma camera and processed with Corridor 4DM software, allows the following conclusions to be drawn:

1. MBF values in studies where automatic image orientation did not require significant manual corrections are characterized by good repeatability, both in assessments by the same and two independent operators.
2. The repeatability of MBF values in case of incorrect automatic image orientation is significantly lower in the RCA vascular territory.
3. The MFR values for the whole myocardium as well as LAD and LCX vascular territories, obtained by the same and two independent operators, are characterized by fairly good repeatability, although it is clearly lower than in PET studies.
4. The repeatability of MFR values in RCA territory obtained by two operators processing studies in the current version of the software is unacceptably poor.
5. Improving the reliability of automatic image orientation and introducing automatic motion correction is likely to significantly improve the repeatability of MFR values and is necessary for a reliable assessment of MFR in RCA territory. This will require further investigation with updated software.
6. In the present situation, patients requiring the assessment of MFR in RCA territory should not undergo the MFR study using the CZT Discovery NM530c camera processed using the version of the Corridor 4DM software used in this paper. PET study results will be much more reliable for this group of patients.

## Figures and Tables

**Table 1:** Age and BMI of the examined patients.

	Min	Max	Median
Age	48	84	64
BMI	17	38	27

**Table 2:** Sex and basic clinical data of the examined patients.

Sex		Post-Infarction	Prior Revascularization		Diabetes
Male	Female		PCI	CABG	
34	23	18	23	0	14

**Table 3:** Spearman’s rank correlation coefficients between MBF values obtained in consecutive assessments by one operator, two weeks apart (1), and by two independent operators (2), with statistical significance of differences between correlations in RCA and other vascular territories. Total number of studies—114 (57 stress and 57 rest studies).

	Operators	TOT	LAD	LCX	RCA	RCA vs. LAD	RCA vs. LCX	RCA vs. TOT
MBF	1	0.97	0.97	0.97	0.94	*p* = 0.0089	*p* = 0.0089	*p* = 0.0089
	2	0.95	0.96	0.95	0.88	*p* < 0.0001	*p* = 0.0008	*p* = 0.0008

**Table 4:** Quality of the automatic image orientation in rest and stress studies.

Automatic Heart Orientation Quality	Stress	Rest	Total
0—little to no adjustments needed	23	5	28
1—axis angle required correction	19	20	39
2—center of axis placed outside of the heart	15	32	47

**Table 5:** Repeatability of the MBF results obtained in two assessments by the same operator (1) and two independent operators (2) in stress and rest studies with varying quality of initial, automatic heart orientation.

Vascular Territory	TOTAL MBF		LAD MBF		LCX MBF		RCA MBF	
Operators	1	2	1	2	1	2	1	2
Good automatic orientation (0) *n* = 28	0.99	0.95	0.98	0.94	0.95	0.95	0.98	0.95
Orientation required adjustments (1, 2) *n* = 86	0.95	0.94	0.95	0.95	0.96	0.93	0.92	0.84
Statistical significance of difference (*p*)	0.0005	0.68	0.04	0.68	0.62	0.45	0.0024	0.0086

**Table 6:** Spearman’s rank correlation coefficients between MFR values obtained in consecutive assessments by one operator, two weeks apart (1) and by two independent operators (2), together with mean differences and SD of MFR values obtained by two operators. Statistical significance of differences between correlations and SD in RCA and other vascular territories are also presented.

	Operators	TOT	LAD	LCX	RCA	RCA vs. LAD	RCA vs. LCX	RCA vs. TOT
MFR	1	0.92	0.88	0.91	0.84	*p* = 0.42	*p* = 0.11	*p* = 0.06
	2	0.89	0.91	0.90	0.67	*p* = 0.0003	*p* = 0.0008	*p* = 0.0019
Mean difference of MFR		0.13	0.10	0.10	0.15			
SD of difference		0.33	0.29	0.37	0.55	*p* < 0.0001	*p* = 0.0036	*p* = 0.0002

## Data Availability

The data presented in this study are available on request from the corresponding author.
